# Asthma, corticosteroid use and schizophrenia: A nationwide population-based study in Taiwan

**DOI:** 10.1371/journal.pone.0173063

**Published:** 2017-03-28

**Authors:** Wei-Chen Wang, Mong-Liang Lu, Vincent Chin-Hung Chen, Mei-Hing Ng, Kuo-You Huang, Ming-Hong Hsieh, Meng-Jer Hsieh, Roger S. McIntyre, Yena Lee, Charles Tzu-Chi Lee

**Affiliations:** 1 School of Medicine, Chung Shan Medical University, Taichung, Taiwan; 2 Department of Psychiatry, Wan Fang Hospital and School of Medicine, College of Medicine, Taipei Medical University, Taipei, Taiwan; 3 Department of Psychiatry, Chang Gung Medical Foundation, Chiayi Chang Gung Memorial Hospital, Chiayi, Taiwan; 4 School of Medicine, Chang Gung University, Taoyuan, Taiwan; 5 Department of Health, Tsaotun Psychiatric Center, Nantou, Taiwan; 6 Department of Speech, Language Pathology and Audiology, Chung Shan Medical University, Taichung, Taiwan; 7 Department of Psychiatry, Chung Shan Medical University Hospital, Taichung, Taiwan; 8 Department of Respiratory Therapy, Chang-Gung University, Taoyuan, Taiwan; 9 Department of Pulmonary and Critical Care Medicine, Chang Gung Medical Foundation, Chiayi Chang-Gung Memorial Hospital, Chiayi, Taiwan; 10 Mood Disorders Psychopharmacology Unit, University Health Network, University of Toronto, Toronto, Ontario, Canada; 11 Department of Psychiatry and Pharmacology, University of Toronto, Toronto, Ontario, Canada; 12 Department of Health Promotion and Health Education, National Taiwan Normal University, Taipei, Taiwan; Kaohsiung Medical University Hospital, TAIWAN

## Abstract

**Objective:**

Asthma and corticosteroid use have been implicated as possible risk factors for schizophrenia. The retrospective cohort study herein aimed to investigate the association between asthma, corticosteroid use, and schizophrenia.

**Method:**

Longitudinal data (2000 to 2007) from adults with asthma (n = 50,046) and without asthma (n = 50,046) were compared on measures of schizophrenia incidence using Taiwan’s National Health Insurance Research Database (NHIRD). Incidence of schizophrenia diagnosis (ICD-9 codes 295.XX) between 2000 and 2007 were compared between groups. Competing risk-adjusted Cox regression analyses were conducted, adjusting for sex, age, residence, socioeconomic status, corticosteroid use, outpatient and emergency room visit frequency, Charlson comorbidity index, and total length of hospital stays days for any disorder.

**Results:**

Of the 75,069 subjects, 238 received a diagnosis of schizophrenia. The mean (SD) follow-up interval for all subjects was 5.8 (2.3) years. After adjusting for potential confounding factors, asthma was associated with significantly greater hazard ratio for incident schizophrenia 1.40 (95% CI = 1.05, 1.87). Additional factors associated with greater incidence of schizophrenia were rural residence, lower economic status, and poor general health. Older age (i.e. ≥65 years) was negatively associated with schizophrenia incidence. Corticosteroid use was not associated with increased risk for schizophrenia.

**Conclusions:**

Asthma was associated with increased risk for schizophrenia. The results herein suggest that a convergent disturbance in the immune-inflammatory system may contribute to the pathoetiology of asthma and schizophrenia.

## Introduction

Schizophrenia is a severe and chronic mental disorder that affects approximately one percent of the general population globally [1]. Schizophrenia is significantly associated with excess and premature mortality, higher rates of medical comorbidity, and deficits in cognitive and psychosocial functioning [2, 3]. Several hypotheses have been proposed to explain the neurodegenerative and abnormal neurodevelopmental processes that subserve the pathogenesis of schizophrenia [4, 5]. One hypothesis posits that inflammatory disturbances may contribute to the etiology of schizophrenia.

Complex interactions between the immune system and the brain have been implicated in the pathoetiology of several psychiatric disorders [6]. For example, several epidemiological studies have reported on an increased risk for schizophrenia among those with autoimmune disorders and/or severe infections [7, 8]. The association between schizophrenia and autoimmune/infectious disorder suggests that there may be a convergent neurobiological substrate [9]. In addition, peripheral inflammation has been associated with greater permeability of the blood—brain barrier, facilitating the entry of immune molecules into the brain [10]. A disturbance in innate and adaptive immunity might contribute to the pathogenesis of schizophrenia [11].

Studies have reported a strong positive correlation between asthma and other mental illnesses such as bipolar disorder and dementia [12–14]. However, only two previous reports (a single case-control design and separate cohort study) have documented an association between asthma and schizophrenia [15, 16]. Both of the foregoing studies reported on a positive correlation between schizophrenia and asthma. Similarly, other studies have reported that asthma patients more likely to develop psychosis experience than non-asthma patients [17–20]. In addition, some reports have reported on the potential impact of corticosteroids, which are commonly used by asthma patients, on the emergence of psychosis [19, 21]. However, no previous study, to our knowledge, has investigated the association between corticosteroid use and schizophrenia. Herein, we investigate the association between the incidence of schizophrenia among individuals with asthma, as well as the possible association between corticosteroid therapy and the incidence of schizophrenia, within a large, retrospective cohort study.

## Materials and methods

### Participants

A retrospective cohort study was assembled using data from the Taiwan National Health Insurance Research Database (NHIRD) provided by the National Health Research Institute (NHRI). The NHIRD includes longitudinal data since its establishment in March 1997 from outpatient, ambulatory, hospital inpatient care, as well as dental services, covered by the National Health Insurance (NHI) program, which includes approximately 98% of Taiwan’s national population. In cooperation with the Bureau of NHI, the NHRI extracted a systematically sampled and nationally representative database of 1,000,000 people from the registry of all NHI enrollees to create the Longitudinal Health Insurance Database (LHID). There were no statistically significant differences in age, sex, or health care utilization costs between the sample comprising the LHID and all enrollees of the NHI [22].

**Fig 1:**
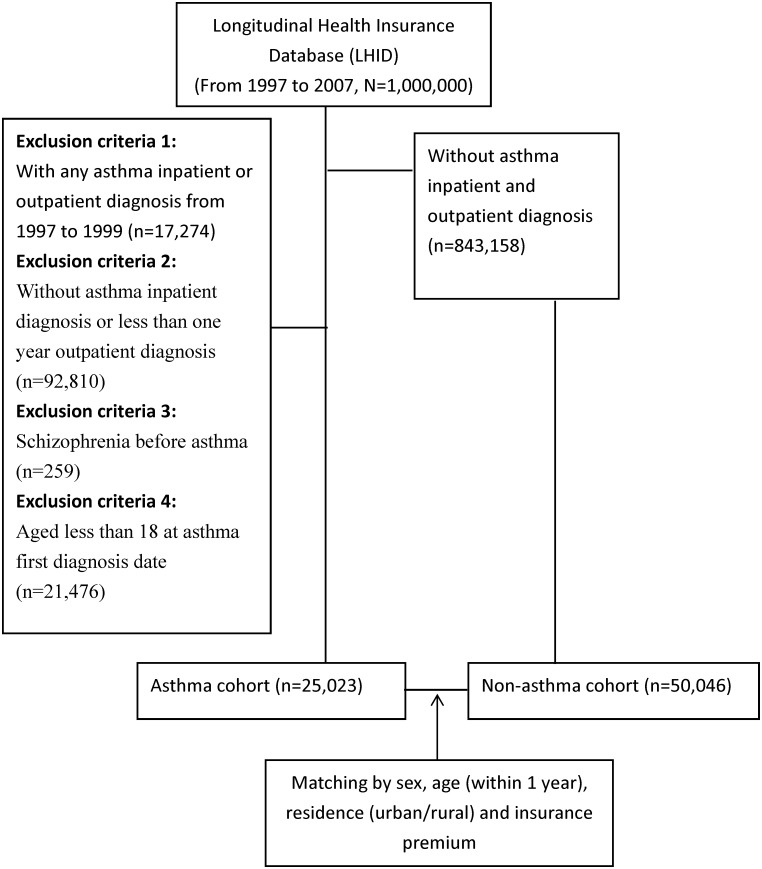
Flowchart of the patient selection process.

Incidence of schizophrenia diagnosis was measured and compared between individuals with and without asthma during the study period (i.e. 1997 to 2007). A diagnosis was operationalized as having a medical claim with the relevant diagnostic code on one occasion through inpatient services or on multiple occasions spread over at least one year through outpatient services; this operationalization is consistent with other research publications using the LHIRD [23]. Asthma was operationalized using the International Classification of Disease, Ninth revision (ICD-9) codes 493.XX. A diagnosis of schizophrenia was operationalized using the ICD-9 codes 295.XX. To assess the incidence of asthma within the study cohort, we excluded data from individuals with a diagnosis of asthma between 1997 and 1999 to make sure our asthma patients were new cases. We excluded data from individuals with a diagnosis of schizophrenia between 1997 and 1999 or with a diagnosis of schizophrenia prior to the diagnosis of asthma. Individuals without asthma (i.e. controls) were randomly sampled from the LHID and matched to individuals with asthma (i.e. cases) on measures of sex, age (+/- 1 year), residence (urban/rural) and insurance premium category. The method of data collection is illustrated in Fig 1.

Covariates considered in this analysis included age, sex, area of residence (urban/rural), economic status, corticosteroid use, outpatient visits, emergency room visits, Charlson comorbidity index and length of the total length of hospital stays for any disorder. In addition, the insurance premium class was classified into three categories and used to proxy economic status: lower income (i.e. requiring social welfare support, no stable salary); middle income (less than 20,000 New Taiwan Dollars [1 USD = 32.1 NTD in 2008] per month); and higher income (20,000 NTD or greater per month).

Corticosteroid use was operationalized and dichotomized as having (or not having) a prescription for corticosteroid for a minimum duration of one year; dose of corticosteroid use was quantified using the annual average cumulative defined daily dose (DDD), defined by the World Health Organization as the dispensed sum of DDDs within one year.

To assess the general physical health of participants during the study period, we used the number of outpatient visits and emergency room visits, total length of hospitalization(s), and Charlson comorbidity index. The foregoing variables were also considered as proxy severity of underlying inflammation. The Charlson comorbidity index comprises a summation of diseases [24].

The NHIRD consists of anonymized data released to the public for research purposes. This study was approved by the Institutional Review Board of Chung Shan Medical University Hospital.

### Statistical analysis

For cases, the index date was operationalized as the date of the incident diagnosis of asthma. The same index date was assigned to the respective matched controls. The outcome of analysis was an incident diagnosis of schizophrenia. The risks for schizophrenia were calculated using survival analyses, with the time function calculated as the number of years from the index date to December 31, 2008 (end of study period) or date of death/ migration (if applicable and earlier than December 31, 2008).

Death prior to incidence of schizophrenia was considered a competing risk event. The death date was retrieved from the national mortality database. The death-adjusted cumulative incidences of schizophrenia were calculated using the Fine and Gray method [25]. Competing risk-adjusted Cox regression models [25] were fitted to evaluate the associations between asthma and schizophrenia incidence, adjusting for covariates. We calculated the hazard ratios with 95% confidence intervals. The model was tested first with all the sample cases included; we subsequently conducted subgroup analyses stratified by sex and age. All data were analyzed using SAS 9.3 software (SAS Institute Inc., Cary, NC, USA). To calculate cumulative incidence and Cox models in the competing risk analysis, we used the R package “cmprsk”[26].

## Results

### Characteristics of subjects

We identified 25,023 individuals with asthma and 50,046 matched controls in the LHID. Study cohort characteristics are described in Table 1. The mean (SD) follow-up interval for all subjects was 5.8 (2.3) years. Of the total 75,069 subjects, 238 were diagnosed with schizophrenia during the study period: 100 (0.40%) of subjects were in the asthma cohort and 138 (0.28%) in the non-asthma cohort. 2,918 (11.66%) individuals with asthma used corticosteroid during the study period, compared to 904 (1.81) individuals without asthma. The annual average cumulative DDD of asthma group 16.17±48.91 was greater than non-asthma group as 3.20±16.71. Individuals with asthma were also more likely than individuals without asthma to have poorer outcomes on measures of general health quality as assessed by the mean of outpatient visits, emergency room visits, Charlson comorbidity indices and total length of hospital stays.

**Table 1:** Characteristics of asthma cases and their matched ^a^ controls.

Characteristic	Asthma		Non-asthma ^a^		p value
	n	%	n	%	
Sex					
Female	13383	53.48	26766	53.48	>0.99
Male	11640	46.52	23280	46.52	
Age group					
18–44	8347	33.36	16692	33.35	0.951
45–64	8656	34.59	17363	34.69	
≥65	8020	32.05	15991	31.95	
Residence					
Urban	17876	71.44	35752	71.44	>0.99
Rural	7147	28.56	14294	28.56	
Insurance premium					
Fixed premium and dependent	7163	28.63	14326	28.63	>0.99
Less than NTD ^b^ 20,000	5777	23.09	11554	23.09	
NTD20,000 or more	12083	48.29	24166	48.29	
Corticosteroid (DDD ^c^)					
0–29	22105	88.34	49142	98.19	<0.001
30–59	1447	5.78	463	0.93	
≥60	1471	5.88	441	0.88	
Mean±SD	16.17±48.91		3.20±16.71		<0.001
Schizophrenia					
No	24923	99.6	49908	99.72	0.005
Yes	100	0.40	138	0.28	
Outpatient visits ^d^					
0–10	314	1.25	4875	9.74	<0.001
11–20	4928	19.69	17687	35.34	
≥21	19781	79.05	27484	54.92	
Mean±SD	27.76±22.56		16.73±17.12		<0.001
Emergency room visits ^d^					
0	22695	90.70	47358	94.63	<0.001
1–2	2050	8.19	2476	4.95	
≥3	278	1.11	212	0.42	
Mean±SD	0.15±0.67		0.07±0.41		<0.001
Charlson comorbidity index ^e^ (Mean±SD)	4.38±3.08		2.68±2.93		<0.001
Total length of hospital stays ^e^ (Mean±SD)	37.94±131.29		18.39±86.42		<0.001

^a^ Matched by sex and age (±1 years old), residence, and insurance premium

^b^ 1US $ = 32.1 NTD in 2008

^c^ DDD: defined daily dose (mg)

^d^ Past one year of index date.

^e^ Past of index date.

### Association between asthma and risk of schizophrenia

**Fig 2:**
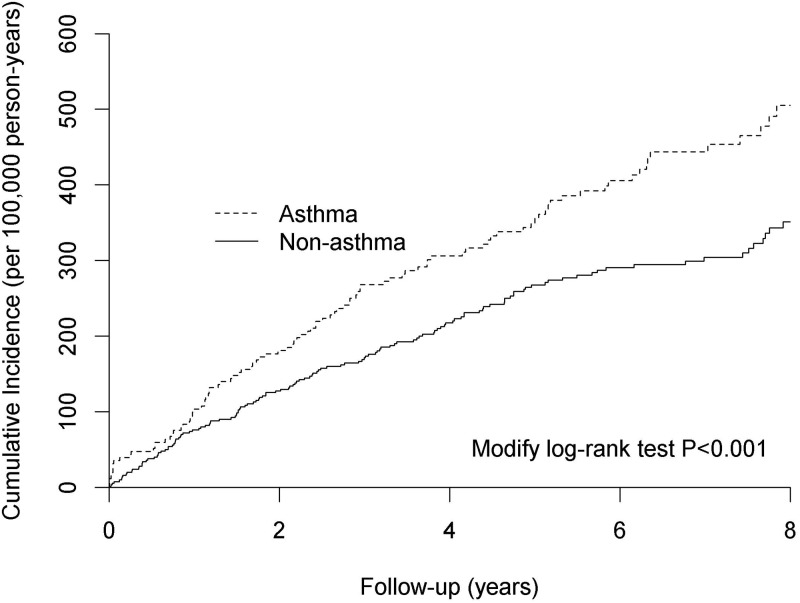
Cumulative incidence of schizophrenia by study group (asthma versus non-asthma).

Analyses of associations of interest are presented in Table 2 and Fig 2. In the fully adjusted Cox regression model for competing risk analysis (i.e. sex, age group, residence, insurance premium, corticosteroid, outpatient visits, emergency room visits, Charlson comorbidity index, the total length of hospital stays and mortality), asthma was positively associated with schizophrenia. Additional factors associated with the incidence of schizophrenia were rural residence, lower economic status, and poor general health. Older age (i.e. ≥65 years) was negatively associated with schizophrenia incidence.

**Table 2:** Competing risk adjusted Cox regression analysis of schizophrenia incidence.

Variable	Unadjusted hazard ratio		Adjusted ^a^ hazard ratio	
	Estimate (95% CI)	P value	Estimate (95% CI)	P value
Asthma				
No	1.00		1.00	
Yes	1.43 (1.10–1.84)	0.007	1.40 (1.05–1.87)	0.020
Sex				
Male	1.00		1.00	
Female	1.20 (0.93–1.55)	0.167	1.17 (0.89–1.54)	0.265
Age group				
18–44	1.00		1.00	
45–64	1.06 (0.82–1.38)	0.649	0.88 (0.64–1.21)	0.434
≥65	0.73 (0.55–0.98)	0.036	0.60 (0.39–0.89)	0.002
Residence				
Urban	1.00		1.00	
Rural	1.31 (1.00–1.71)	0.046	1.55 (1.17–2.04)	0.002
Insurance premium				
Fixed premium and dependent	1.00		1.00	
Less than NTD ^b^ 20,000	2.15 (1.66–2.80)	<0.001	1.43 (1.04–1.97)	0.030
NTD20,000 or more	0.46 (0.35–0.60)	<0.001	0.50 (0.35–0.70)	<0.001
Corticosteroid (100 DDD ^c^)	1.16 (0.94–1.33)	0.178	1.17 (0.95–1.39)	0.250
Outpatient visits ^d^				
0–10	1.00		1.00	
11–20	0.51 (0.32–1.08)	0.105	0.59 (0.36–1.07)	0.137
≥21	0.71 (0.47–1.08)	0.111	0.83 (0.52–1.30)	0.450
Emergency room visits ^d^				
0	1.00		1.00	
1–2	2.15 (1.42–3.25)	<0.001	2.11 (1.38–3.24)	<0.001
≥3	2.49 (0.80–7.77)	0.116	2.29 (0.72–7.27)	0.159
Charlson comorbidity index ^e^	0.98 (0.94–1.02)	0.329	0.96 (0.91–1.01)	0.139
Total length of hospital stays ^e^ (100 days)	1.09 (1.07–1.11)	<0.001	1.08 (1.06–1.10)	<0.001

^a^ Competing risk adjusted Cox regression analysis controlling by sex, age, residence, insurance amount, corticosteroid, outpatient visits, emergency room visits, Charlson comorbidity index and total length of hospital stays.

^b^ 1US $ = 32.1 NTD in 2008

^c^ DDD: Defined daily dose (mg)

^d^ Past one year of index date.

^e^ Past of index date.

CI: Confidence interval.

In secondary analyses within the asthma cohort, factors associated with incidence of schizophrenia included: older age (i.e. ≥65 years), rural residence, lower economic status and poor general health (Table 3). In stratified analyses, the association of interest was statistical significance in men and in the 18–64 year age group (Table 4).

**Table 3:** Competing risk adjusted Cox regression analysis of schizophrenia incidence among asthma patients.

Variable	Unadjusted hazard ratio		Adjusted ^a^ hazard ratio	
	Estimate (95% CI)	P value	Estimate (95% CI)	P value
Sex				
Male	1.00		1.00	
Female	1.03 (0.69–1.52)	0.899	1.02 (0.68–1.52)	0.941
Age group				
18–44	1.00		1.00	
45–64	1.14 (0.76–1.71)	0.513	0.90 (0.54–1.49)	0.676
≥65	0.67 (0.42–1.05)	0.083	0.49 (0.26–0.93)	0.030
Residence				
Urban	1.00		1.00	
Rural	1.32 (0.87–1.99)	0.190	1.54 (1.01–2.36)	0.044
Insurance amount				
Fixed premium and dependent	1.00		1.00	
Less than NTD ^b^ 20,000	2.23 (1.49–3.32)	<0.001	1.55 (0.95–2.52)	0.077
NTD20,000 or more	0.48 (0.32–0.74)	0.001	0.54 (0.32–0.92)	0.023
Corticosteroid (100 DDD ^c^)	1.13 (0.91–1.45)	0.111	1.12 (0.90–1.56)	0.356
Outpatient visits ^d^				
0–10	1.00		1.00	
11–20	0.34 (0.10–1.15)	0.082	0.41 (0.12–1.42)	0.160
≥21	0.42 (0.13–1.32)	0.138	0.56 (0.17–1.82)	0.335
Emergency room visits ^d^				
0	1.00		1.00	
1–2	2.34 (1.35–4.05)	0.002	2.27 (1.28–4.02)	0.005
≥3	2.41 (0.59–9.78)	0.219	2.24 (0.55–9.10)	0.260
Charlson comorbidity index ^e^	0.98 (0.92–1.04)	0.509	0.96 (0.90–1.04)	0.319
Total length of hospital stays ^e^ (100 days)	1.08 (1.05–1.10)	<0.001	1.07 (1.04–1.09)	<0.001

^a^ Competing risk adjusted Cox regression analysis controlling by sex, age, residence, insurance amount, corticosteroid, outpatient visits, emergency room visits, Charlson comorbidity index and total length of hospital stays.

^b^ 1US $ = 32.1 NTD in 2008

^c^ DDD: Defined daily dose (mg)

^d^ Past one year of index date.

^e^ Past of index date.

CI: Confidence interval.

**Table 4:** Association between asthma and schizophrenia incidence by sex and age group.

Subgroup	Adjusted hazard ratio for schizophrenia ^a^	
	Estimate (95% CI)	P value
**Total sample**	1.40 (1.05–1.87)	0.020
**Sex**		
Male	1.59 (1.01–2.52)	0.047
Female	1.30 (0.90–1.87)	0.169
**Age group**		
18–64	1.47 (1.03–2.12)	0.044
≥65	1.25 (0.69–2.32)	0.128

^a^ Competing risk adjusted Cox regression analysis controlling by sex, age, residence, insurance amount, corticosteroid, outpatient visits, emergency room visits, Charlson comorbidity index and total length of hospital stays.

CI: Confidence interval.

## Discussion

In our cohort study using the NHIRD, a diagnosis of asthma was significantly associated with a greater risk for schizophrenia (unadjusted hazard ratio = 1.43, 95% CI = 1.10 to 1.84). After adjusting for possibly confounding variables (i.e. sex, age, residence, economic status, corticosteroid use, general health quality), the adjusted hazard ratio was only modestly diminished to 1.40 (95% CI = 1.05–1.87). Corticosteroid use was not associated with greater risk for schizophrenia.

To our knowledge, this is one of few population-based cohort studies to report on an association between asthma and schizophrenia. In addition, to our knowledge, no previous study has reported on the moderation role of corticosteroid use on the foregoing association. Our results replicate findings from previous studies [15, 16] For example, Chen et al. [15] reported that, within a cross-sectional dataset, patients with schizophrenia had a 1.3-fold increased risk for concurrent asthma. A separate longitudinal study using [16] two nationwide population-based registers in Denmark reported that asthma is associated with increased risk for schizophrenia (HR = 1.59, 95% CI = 1.31–1.90).

The association of corticosteroid use and psychosis has been reported in several previous studies [19, 21]. However, our findings did not find a statistically significant association between corticosteroid use and schizophrenia among asthma population. Bag et al. [21], showed a case report with steroid-induced psychosis in a 12-year-old boy and this may not be diagnosed as schizophrenia in clinical practice. Drozdowicz LB et al. [19], reported the review regrading psychiatric adverse effects of pediatric corticosteroid use. They found most of the evidence is still at the level of case reports, case series, and small trials. The psychosis experience, such as paranoid ideation, cannot be diagnosed as schizophrenia. Since the limitation of previous reports and most of them described the psychosis symptoms rather than schizophrenia, the association of corticosteroid use and schizophrenia is still unknown. We used the population-based cohort study to demonstrate that corticosteroid use is not linked to schizophrenia.

Immune-inflammatory processes may subserve the pathogenesis of asthma and schizophrenia [27–29] Emerging evidence suggests that infectious/autoimmune processes play an important role in the etiopathogenesis of subpopulations with schizophrenia [30, 31]. Among inflammatory peptides, cytokines are believed to contribute to the pathophysiology of many psychiatric illnesses, especially schizophrenia [32–36]. Available evidence indicates that abnormalities in the balance of pro- and anti-inflammatory cytokines in both central and peripheral compartments contribute to the abnormal neural structure and function reported in schizophrenia.

### Limitations and strengths

This is one of few cohort studies using a nationally representative sample and longitudinal dataset to investigate the association between asthma and schizophrenia as well as the possible moderation role of corticosteroid use. The method of using the national database from NHIRD limits selection bias which is demonstrated from the similar prevalence of schizophrenia from the present study (0.27%) to that (0.2–0.3%) from community survey [37]. Furthermore, in our cohort study, we reduced the possibility of reverse casualty by using a longitudinal design in which the diagnosis of asthma predated the diagnosis of schizophrenia.

There are important limitations to our methodology that may limit inferences that may be made from our findings. The NHIRD did not contain data on possibly confounding variables such as smoking, dietary habits, family history and maternal condition. Another limitation is that although our data are based on physicians’ diagnoses, we cannot control for variability between clinicians in establishing a clinical diagnosis of asthma. Moreover, the severity of schizophrenia and asthma are unknown and could not be evaluated directly in the analysis herein. In epidemiological studies, the time of first symptom onset of asthma is most relevant. However, our study database did not contain information regarding first symptom onset of asthma.

## Conclusions

The study suggests that the clinical care of patients with an asthma diagnosis necessitates monitoring for emerging psychopathology including, but not limited to schizophrenia. The future research vista is to identify convergent etipoathogenic mechanism subserving and or mediation the association between asthma and schizophrenia e.g immuno-inflammatory systems.
